# Ovo-Like Transcriptional Repressor 1 (OVOL1) Expression in Pleomorphic Adenoma and Carcinoma Ex Pleomorphic Adenoma: Diagnostic and Prognostic Insights

**DOI:** 10.7759/cureus.94331

**Published:** 2025-10-11

**Authors:** Aasha Mohamed, Nadia Abbas El-Sissy, Osama Abdelrahim El Kashty, Marwa M. Zaki

**Affiliations:** 1 Department of Oral and Maxillofacial Pathology, Faculty of Dentistry, Zagazig University, Zagazig, EGY; 2 Department of Oral Pathology, Faculty of Dentistry, Mansoura University, Mansoura, EGY; 3 Department of Pathology, Faculty of Medicine, Mansoura University, Mansoura, EGY

**Keywords:** carcinoma ex pleomorphic adenoma, clinicopathological, immunohistochemical, ovol1, pleomorphic adenoma

## Abstract

Introduction

Pleomorphic adenoma (PA) is the most common benign tumor of the salivary glands and has the potential for malignant transformation into carcinoma ex pleomorphic adenoma (CXPA). Ovo-like transcriptional repressor 1 (OVOL1) is a transcription factor that regulates epithelial differentiation by suppressing epithelial-mesenchymal transition (EMT) and promoting mesenchymal-epithelial transition (MET). OVOL1 expression is regulated by the Wnt signaling pathway, a central regulator of epithelial stem cell development, maintenance, and differentiation. While several transcription factors are implicated in EMT/MET, OVOL1 is of particular interest because of its potential dual role in maintaining epithelial identity and modulating tumor behavior. Its dysregulation has been associated with adverse prognosis in several cancers. Therefore, this study aims to investigate OVOL1 expression in PA and CXPA to clarify its potential role in the transition from benign to malignant lesions and to improve the assessment of tumor biological behavior.

Materials and methods

This retrospective comparative cohort study included 40 cases, 20 cases of PA and 20 cases of CXPA, diagnosed between 2017 and 2024, retrieved from the archive of the Pathology Laboratory at Mansoura University’s Oncology Center. Owing to the rarity of CXPA, all eligible cases during the study period were included. To provide a balanced comparator group and minimize selection bias, an equal number of PA cases were consecutively selected from the same archive and timeframe. Clinical data were obtained from electronic medical records. Hematoxylin and eosin-stained slides were reviewed by expert pathologists to confirm the diagnosis. Morphological subtyping of CXPA was performed according to the most recent WHO Classification of head and neck tumors. OVOL1 immunohistochemical expression was evaluated in both PA and CXPA using a combined semi-quantitative immunoreactive score (IRS) and quantitative digital image analysis (ImageJ software), ensuring greater objectivity and reproducibility. Correlations with clinicopathological features were examined using appropriate statistical tests.

Results

All studied PA and CXPA cases were OVOL1-positive with different levels of expression. PA cases predominantly showed high expression, while most CXPA demonstrated low levels, with a significant difference between the two groups. Low OVOL1 expression was more frequent in high-grade and recurrent CXPA, suggesting a link with tumor aggressiveness.

Conclusion

Our results suggest the potential of OVOL1 as a diagnostic and prognostic biomarker, particularly in distinguishing PA from CXPA, and as a promising indicator of tumor aggressiveness and malignant transformation. While these findings are limited by the small cohort size, they provide preliminary evidence that warrants validation in larger, multi-institutional studies exploring their clinical significance and underlying molecular mechanisms.

## Introduction

Pleomorphic adenoma (PA) is the most common benign salivary gland tumor [1]. It is characterized by diverse morphology, comprising an admixture of ductal, myoepithelial, and stromal elements. The cellular phenotype, particularly of the myoepithelial cells, exhibits a wide morphological range [2].

Malignant transformation of PA into carcinoma ex pleomorphic adenoma (CXPA) occurs in approximately 6% of cases [3]. This is typically a late phenomenon, usually occurring many years after the initial diagnosis or in recurrences following inadequate excision. CXPA is characterized by malignant epithelial and/or myoepithelial components associated with a primary or recurrent PA [4]. Diagnosis requires identification of both benign and malignant components; however, if the benign component has been completely replaced by carcinoma, prior clinical or histological documentation of PA at the same site is necessary [2].

The morphology of the carcinomatous component includes salivary duct carcinoma (SDC), which is the most common form, followed by myoepithelial carcinoma (MC), epithelial-myoepithelial carcinoma (EMC), and adenocarcinoma not otherwise specified (Adeno-NOS) [5].

High cellularity, cellular pleomorphism, and abnormal mitotic activity are the most common features of malignant transformation in PA [6]. Solid cellular PA may also display increased cellularity and mild nuclear atypia, which can mimic low-grade CXPA. However, unlike CXPA, solid PA typically lacks significant mitotic activity, infiltrative growth, and destructive invasion into adjacent tissues. Therefore, differentiating solid cellular PA from low-grade CXPA remains one of the most challenging problems in salivary gland pathology, requiring careful evaluation of mitotic figures, destructive invasion, and overtly malignant cytological features [7].

Epithelial-mesenchymal transition (EMT) is one of the key mechanisms underlying the transformation of benign into malignant neoplasms. The ovo-like transcriptional repressor 1 (OVOL1), a member of the zinc finger protein family of transcription factors, is a critical regulator of both normal epithelial and cancer cell differentiation [8]. OVOL1 can inhibit EMT while promoting mesenchymal-epithelial transition (MET). OVOL1 expression is regulated by the Wnt signaling pathway, a central regulator of epithelial stem cell development, maintenance, and differentiation. Its dysregulation has been associated with adverse prognosis in several cancers [9].

Although malignant transformation of benign salivary gland tumors has been documented in the literature, the process remains poorly understood in PA [7]. Accordingly, a detailed investigation of OVOL1 expression in PA and CXPA is expected to contribute to a better understanding of the underlying mechanisms of tumor progression and to improve the assessment of tumor biological behavior. Therefore, this study aimed to investigate OVOL1 expression in PA and CXPA, to clarify its role in the transition from benign to malignant lesions, and to analyze its potential diagnostic and prognostic significance.

## Materials and methods

Study design

This retrospective comparative cohort study included 40 cases, 20 cases of PA and 20 cases of CXPA, diagnosed between 2017 and 2024, and was retrieved from the archive of the Pathology Laboratory at Mansoura University’s Oncology Center. Given the rarity of CXPA, all eligible cases meeting the inclusion criteria during the study period were incorporated. To provide a balanced comparator group, an equal number of PA cases were selected consecutively from the same archive and time frame to ensure balance and reduce selection bias. The study included cases with available comprehensive clinical and histopathological data, and formalin-fixed paraffin-embedded (FFPE) tissue blocks sufficient for immunohistochemical staining. Cases with incomplete clinical data or inadequate or unavailable tissue blocks were excluded from the study.

Clinical characteristics and histopathological evaluation

Patients’ data were reviewed from electronic medical records. The pathology reports and hematoxylin and eosin (H&E) stained slides of the included cases were independently reviewed and reassessed by two expert pathologists to confirm the diagnosis and to evaluate the clinicopathological parameters, including patient age, gender, anatomical site, recurrence status, and tumor, lymph node, and metastasis (TNM) stage (for CXPA cases). Morphological subtyping of CXPA was performed in accordance with the most recent WHO Classification of Head and Neck Tumors, 5th Edition [5].

Immunohistochemical staining protocol

Immunostaining was performed on 4 μm sections from FFPE tissue blocks. Endogenous peroxidase activity was blocked using 3% hydrogen peroxide. Antigen retrieval was carried out in Tris-EDTA citrate buffer solution (pH 9.0). Sections were then incubated with OVOL1 antibody (rabbit polyclonal, anti-human OVOL1; dilution 1:100; catalog no. ARC0038, Medaysis, USA). The antigen-antibody complex was detected using a prediluted horseradish peroxidase-labeled reagent, followed by counterstaining with 0.02% hematoxylin. Positive and negative controls were run in parallel. Kidney tissue was used as a positive control for OVOL1 immunostaining, while the negative control was prepared by omitting the primary antibody to ensure specificity.

Immunohistochemical interpretation

The interpretation of immunohistochemical staining was performed semiquantitatively and independently by two pathologists blinded to the clinical outcomes. OVOL1-positive expression was defined as cytoplasmic and/or membranous staining. The final score was calculated as the product of the staining intensity and percentage scores. Guided by the positive control, staining intensity was graded on a semiquantitative scale from 0 to 3: 0 (negative), 1 (weak), 2 (moderate), and 3 (strong). The percentage of positively stained cells was scored as follows: ≤5% (0), 6-25% (1), 26-50% (2), 51-75% (3), and >75% (4) [10]. According to the immune reactive score (IRS) described by Harb et al., a total score of 0-3 was considered low expression, whereas a score of 4-12 was considered high expression [11].

To minimize intra- and interobserver variability, both pathologists performed the scoring independently, and discrepant cases were resolved by joint review to reach consensus. To enhance reproducibility and provide objective quantification, digital image analysis was subsequently conducted using ImageJ software on all cases. Standardized parameters were applied to measure integrated density, mean intensity, and the fractional proportion of stained pixels within defined regions of interest. IRS served as the primary pathology-based classification for categorical analyses, while ImageJ outputs were used as supportive quantitative validation. In instances of divergence, slides were re-examined jointly by the pathologists with ImageJ data available as supplementary evidence, and the final classification was determined by consensus. This combined approach integrated expert morphological assessment with computer-assisted quantification, reinforcing the robustness and reliability of the results.

Statistical analysis

Statistical analysis was performed using IBM SPSS Statistics for Windows, Version 25 (Released 2017; IBM Corp., Armonk, New York, United States). Categorical variables were summarized as frequencies and percentages. Associations between clinicopathological variables and immunohistochemical marker expression were primarily assessed using Pearson's Chi-square (χ2) test for independence. However, for tables where the Chi-square assumption was violated (i.e., when more than 20% of the expected cell frequencies were less than 5), Fisher’s Exact Test was applied for 2 × 2 tables, and the Fisher-Freeman-Halton Exact Test was used for larger contingency tables, to ensure a reliable p-value.

Results are presented with χ2 values, degrees of freedom (df) (for standard Chi-square tests), effect size, and corresponding p-values. Effect size was calculated using Phi (ϕ) for 2 × 2 tables and Cramér’s V (V) for larger contingency tables. Effect size statistics are reported for all tables, including those analyzed with Fisher’s Exact Test, as the χ2 statistic remains the conventional measure for association strength. Effect size values were interpreted according to Cohen’s guidelines, where 0.1 indicates a small/weak association; 0.3, a medium/moderate association; and ≥0.5, a large/strong association. A p-value of <0.05 was considered statistically significant.

## Results

**Figure 1:**
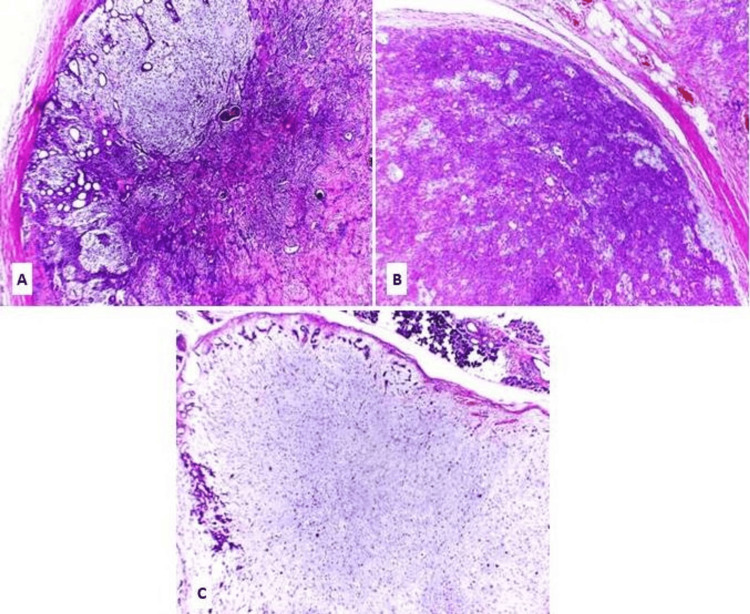
Pleomorphic adenoma H&E: hematoxylin and eosin; PA: pleomorphic adenoma (A) Classic phenotype of PA (H&E, x100). (B) Cellular phenotype of PA (H&E, x100). (C) Stromal phenotype of PA (H&E, x100)

The current study comprised 20 cases of PA and 20 cases of CXPA. Among the PA group, nine cases (45%) exhibited the classic phenotype, six cases (30%) displayed the cellular phenotype, and five cases (25%) demonstrated the stromal phenotype (Figure 1).

**Figure 2:**
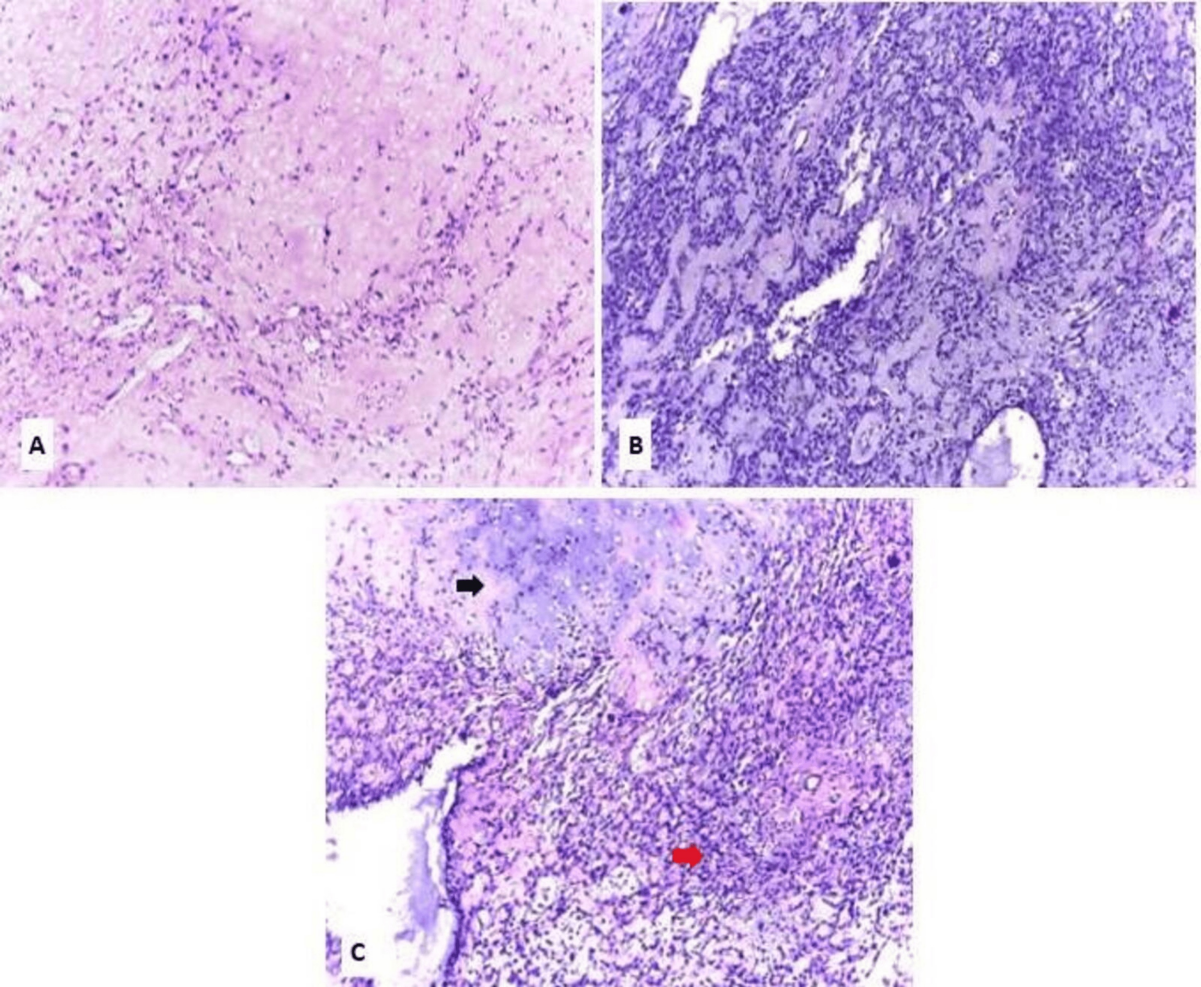
Epithelial myoepithelial carcinoma ex pleomorphic adenoma EMC ex PA: epithelial myoepithelial carcinoma ex pleomorphic adenoma; H&E: hematoxylin and eosin; PA: pleomorphic adenoma (A) Residual benign component of PA with extensive hyalinization (H&E, x100). (B) EMC ex PA, showing biphasic proliferation of inner ductal epithelial cells surrounded by an outer layer of clear myoepithelial cells (H&E, x100). (C) EMC ex PA (red arrow) with adjacent residual benign PA (black arrow) (H&E, x100)

**Figure 3:**
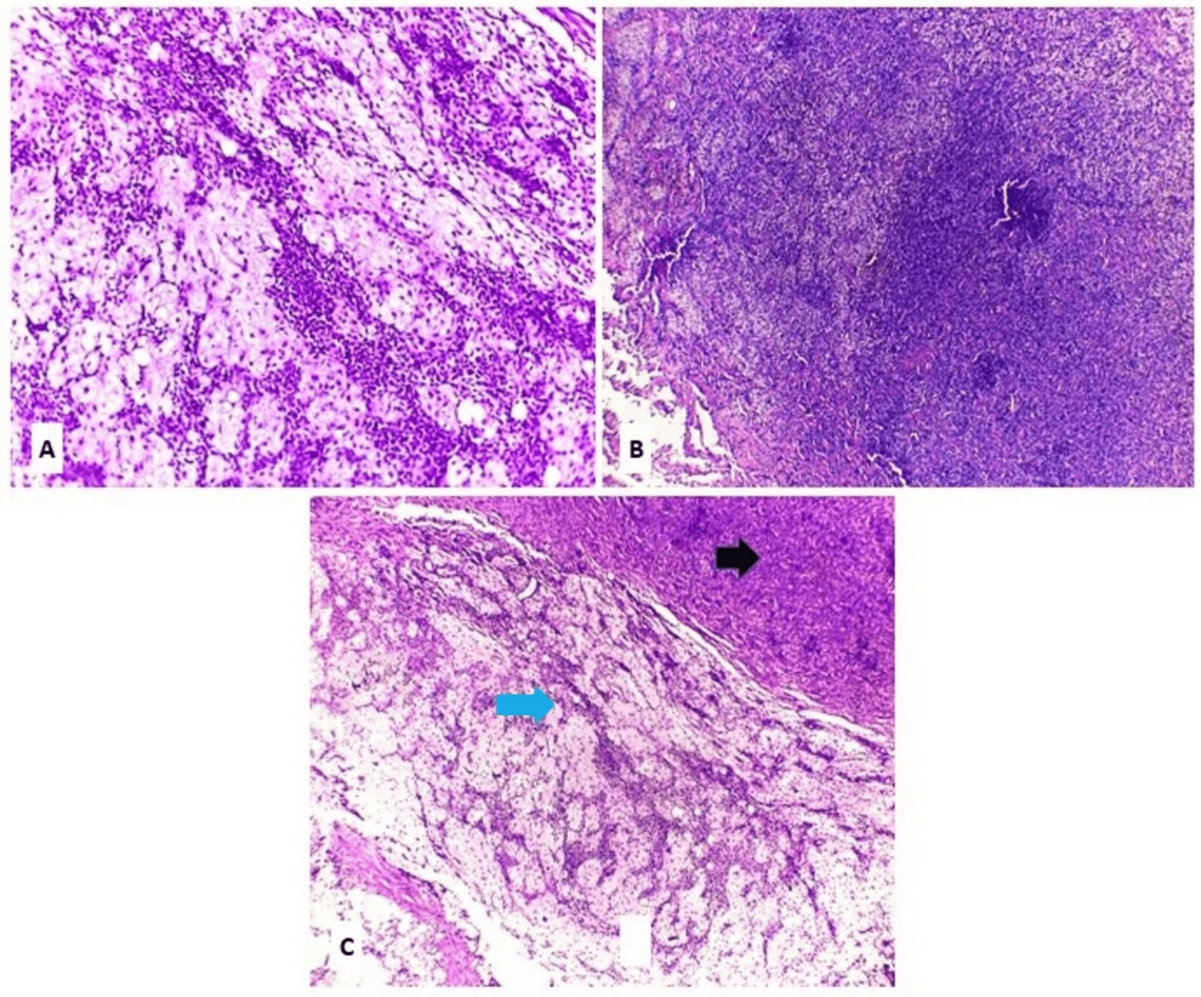
Myoepithelial carcinoma ex pleomorphic adenoma H&E: hematoxylin and eosin; MC ex PA: myoepithelial carcinoma ex pleomorphic adenoma; PA: pleomorphic adenoma (A) Residual PA with a prominent fibromyxoid stroma (H&E X100). (B) MC ex PA composed of sheets and nests of atypical myoepithelial cells with predominantly epithelioid morphology and focal clear cell change (H&E X40). (C) Area of malignant transformation of PA (blue arrow) to MC (black arrow) (H&E X40)

**Figure 4:**
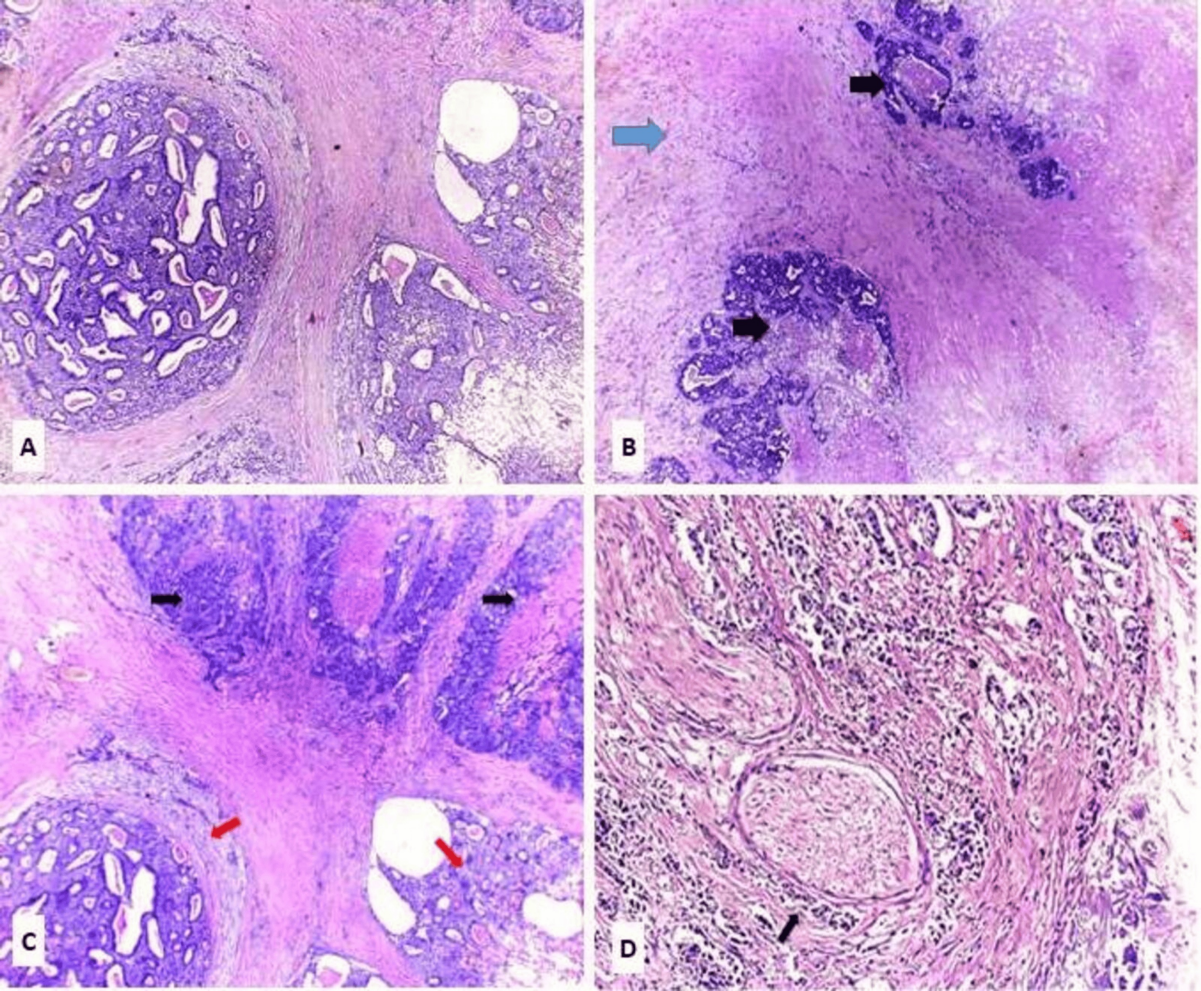
Salivary duct carcinoma ex pleomorphic adenoma H&E: hematoxylin and eosin; PA: pleomorphic adenoma; SDC ex PA: salivary duct carcinoma ex pleomorphic adenoma (A) SDC ex PA, intraductal component formed of tumor cells with luminal spaces resembling a sieve-like architecture (H&E, X40). (B) SDC ex PA, invasive component formed of neoplastic glands and solid nests with necrotic areas (black arrow), infiltrating adjacent residual PA with chondromatous and hyalinized change (blue arrow) (H&E, X40). (C) SDC ex PA, intraductal component (red arrow) and invasive component (black arrow) (H&E, X40). (D) SDC ex PA showing perineural invasion (black arrows) (H&E, X100)

**Figure 5:**
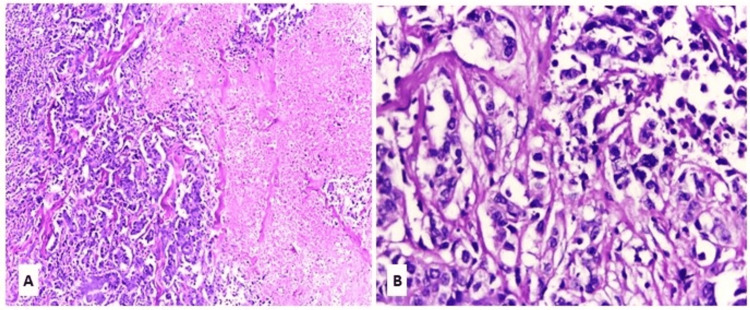
Adenocarcinoma-not otherwise specified ex pleomorphic adenoma Adeno-NOS ex PA: adenocarcinoma-not otherwise specified ex pleomorphic adenoma; H&E: hematoxylin and eosin (A) Adeno-NOS ex PA, high-grade adenocarcinoma composed of pleomorphic, hyperchromatic tumor cells arranged in sheets and nests, with focal attempts at glandular differentiation and extensive areas of necrosis (H&E, x100). (B) Aggregates and irregular glandular formations formed of atypical cells (H&E, x400)

Regarding the CXPA group, the morphological subtyping according to the WHO Classification of Head and Neck Tumors, 5th Edition, revealed six cases (30%) of EMC ex PA, four cases (20%) of MC ex PA, seven cases (35%) of SDC ex PA, and three cases (15%) of Adeno-NOS ex PA (Figures 2-5).

The clinicopathological characteristics of the studied PA and CXPA cases are summarized in Table 1.

**Table 1:** Clinicopathological parameters of the studied cases CXPA: carcinoma ex pleomorphic adenoma; N: total number of cases; PA: pleomorphic adenoma; TNM: tumor, lymph node, and metastasis Note: TNM staging was applied only to CXPA cases

Clinicopathological parameters		PA (N = 20)	CXPA (N = 20)
Age (years)	30-50	13 (65%)	10 (50%)
	51-71	5 (25%)	8 (40%)
	72-91	2 (10%)	2 (10%)
Gender	Males	7 (35%)	14 (70%)
	Females	13 (65%)	6 (30%)
Site	Parotid gland	13(65%)	14 (70%)
	Submandibular glands	4 (20%)	3 (15%)
	Palatal glands	3 (15%)	3 (15%)
Tumor recurrence	Recurrent	1	4
	Nonrecurrent	19	16
TNM staging	Stage I	-	2 (10%)
	Stage II	-	4 (20%)
	Stage III	-	6 (30%)
	Stages IVA	-	8 (40%)

**Figure 6:**
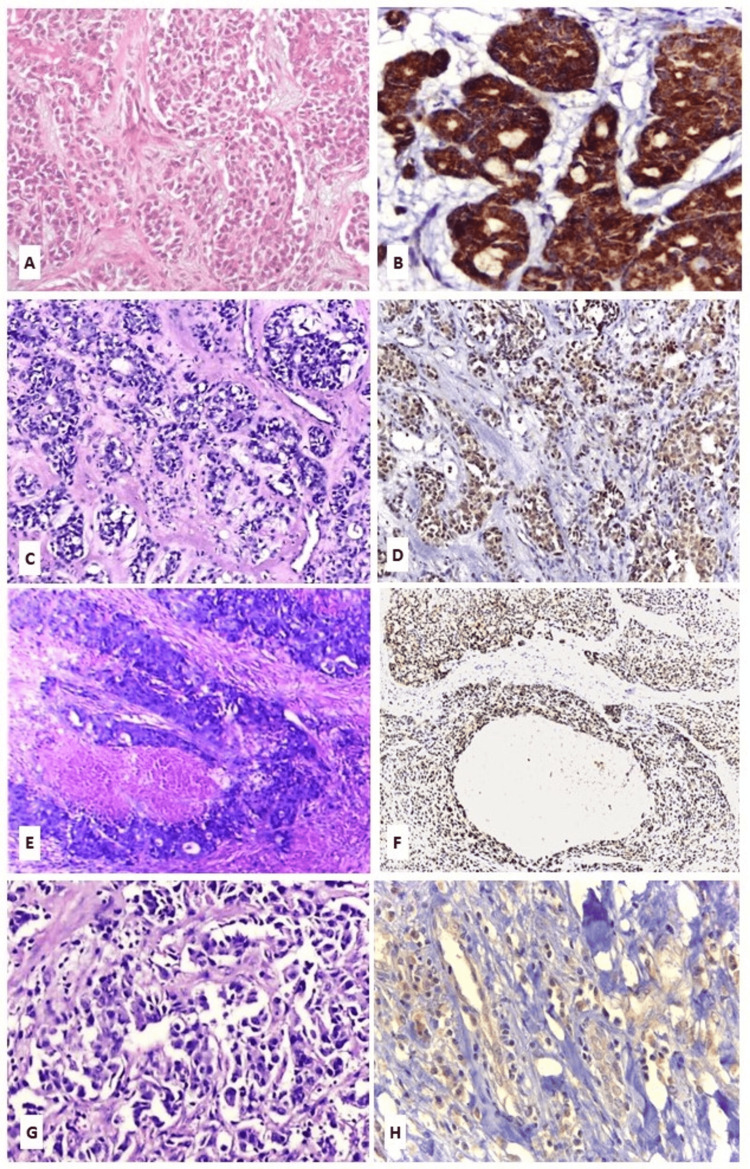
OVOL1 immunohistochemical staining Adeno-NOS ex PA: adenocarcinoma, not otherwise specified ex pleomorphic adenoma; EMC ex PA: epithelial myoepithelial carcinoma ex pleomorphic adenoma; H&E: hematoxylin and eosin; MC ex PA: myoepithelial carcinoma ex pleomorphic adenoma; OVOL1: ovo-like transcriptional repressor 1; PA: pleomorphic adenoma; SDC ex PA: salivary duct carcinoma ex pleomorphic adenoma (A) PA (H&E, x200). (B) PA showing high OVOL1 expression (OVOL1 antibody, x200). (C) EMC ex PA (H&E, x200). (D) EMC ex PA showing low OVOL1 expression (OVOL1 antibody, x200). (E) SDC ex PA (H&E, x200). (F)  SDC ex PA showing low OVOL1 expression (OVOL1 antibody, x200). (G) Adeno-NOS ex PA, high grade (H&E, x400). (H) Adeno-NOS ex PA, high grade, showing low OVOL1 expression (OVOL1 antibody, x400)

As regards OVOL1 expression, positive immunoreactivity was observed in all examined PA and CXPA cases, with variable levels of low and high expressions (Figure 6).

High OVOL1 expression was detected in 12 PA cases (60%) compared with only three CXPA cases (15%). Statistical analysis demonstrated a significant difference between the two groups (χ² = 8.64, p = 0.003), with a large effect size (0.5), indicating a strong association between OVOL1 expression and tumor type (Table 2).

**Table 2:** OVOL1 expression among the studied pleomorphic adenoma and carcinoma ex pleomorphic adenoma cases CXPA: carcinoma ex pleomorphic adenoma; OVOL1: ovo-like transcriptional repressor 1; PA: pleomorphic adenoma; χ²: test statistic for association; df: degree of freedom; *: significant difference (p < 0.05); N: total number of cases

OVOL1 expression	PA (N = 20)	CXPA (N = 20)	χ²	df	Effect size (Phi)	p-value
Low expression	8 (40%)	17 (85%)	8.64	1	0.5 (large)	0.0032*
High expression	12 (60%)	3 (15%)				

Regarding OVOL1 expression among the CXPA cases, our results demonstrated a statistically significant association between OVOL1 expression and the morphological subtypes of CXPA (χ² = 8.0486, p = 0.0354), with a large effect size (0.63), indicating a strong relationship (Table 3).

**Table 3:** Correlation between OVOL1 expression and histopathological types of studied carcinoma ex pleomorphic adenoma cases Adeno-NOS ex PA: adenocarcinoma, not otherwise specified ex pleomorphic adenoma; EMC ex PA: epithelial myoepithelial carcinoma ex pleomorphic adenoma; MC: myoepithelial carcinoma ex pleomorphic adenoma; OVOL1: ovo-like transcriptional repressor 1; SDC: salivary duct carcinoma ex pleomorphic adenoma; χ²: test statistic for association; *: significant difference (p < 0.05); N: total number of cases (a) p-value calculated using the Fisher-Freeman-Halton Exact Test due to low expected cell counts (<5) violating Chi-square assumptions. The χ2 statistic and Cramér’s V are reported as the conventional measures of effect size

OVOL1 expression	EMC ex PA (N = 6)	MC ex PA (N = 4)	SDC ex PA (N = 7)	Adeno-NOS ex PA (N = 3)			χ²	Effect size (Cramér’s V)	p-value
				Low grade	Intermediate grade	High grade			
Low	6 (100%)	4 (100%)	6 (85.7%)	0	0	1 (33.3%)	≈ 8.0486	0.6344 (large)	0.0354* (a)
High	0	0	1 (14.3%)	1 (33.3%)	1 (33.3%)	0			

Concerning the relationship between OVOL1 expression and capsular invasion in CXPA, low expression was observed in all minimally invasive cases and in six cases (40%) with wide capsular invasion. In contrast, high expression was detected in all noninvasive cases and in nine cases (60%) of widely invasive cases. Analysis of the association between OVOL1 expression and the pattern of capsular invasion revealed a statistically significant relationship (χ2 = 5.0, p = 0.005). The calculated effect size (0.5) indicates a large and strong association (Table 4).

**Table 4:** Correlation of OVOL1 expression with the capsular invasion of the studied carcinoma ex pleomorphic adenoma cases CXPA: carcinoma ex pleomorphic adenoma; OVOL1: ovo-like transcriptional repressor 1; χ²: test statistic for association; *: significant difference (p < 0.05); N: total number of cases (a) p-value obtained using the Fisher-Freeman-Halton Exact Test due to low expected cell counts (<5) violating Chi-square assumptions. The χ2 statistic and Cramér’s V are reported as the conventional measures of effect size

Capsular invasion	OVOL1 expression	CXPA (N = 20)	χ²	Effect size (Cramér’s V)	p-value
Non-invasive	Low	0	5.0	0.5 (large)	0.005* (a)
	High	3 (100%)			
Minimal invasive	Low	2 (100%)			
	High	0			
Widely invasive	Low	6 (40%)			
	High	9 (60%)			

Among recurrent cases, high OVOL1 expression was detected in only one case (20%) of PA and in none of the CXPA cases, whereas low expression predominated in recurrent CXPA (4 cases, 80%). The analysis did not demonstrate a statistically significant association (p = 0.2). Although the Chi-square statistic (χ² = 5.0) corresponded to a very strong effect size (1.0), the small sample size in this sub-analysis (N = 5) limited the statistical power, and the result did not reach the conventional significance threshold.

Conversely, in nonrecurrent cases, high OVOL1 expression was more frequent in PA (11 cases, 31.6%) compared with CXPA (3 cases, 8.6%), while low expression was more common in CXPA (13 cases, 37.2%). This association was statistically significant (χ² = 5.546, p = 0.0185). This indicates that OVOL1 expression patterns differ significantly between PA and CXPA in nonrecurrent cases. The strength of this association corresponds to a medium-to-strong effect size (0.398) (Table 5).

**Table 5:** Correlation between OVOL1 expression and tumor recurrence in the studied pleomorphic adenoma and carcinoma ex pleomorphic adenoma cases CXPA: carcinoma ex pleomorphic adenoma; OVOL1: ovo-like transcriptional repressor 1; PA: pleomorphic adenoma; χ²: test statistic for association; df: degree of freedom; *: significant difference (p < 0.05); N: total number of cases (a) p-value obtained using the Fisher-Freeman-Halton Exact Test due to low expected cell counts (<5) violating Chi-square assumptions. The χ2 statistic and Phi are reported as the conventional measures of effect size

Tumor recurrence	OVOL1 expression	PA (N = 20)	CXPA (N = 20)	χ²	df	Effect size (Phi)	p-value
Recurrent	Low	0	4 (80%)	5.0	N/A	1.0 (large)	0.200 (a)
	High	1 (20%)	0				
Non-recurrent	Low	8 (22.8%)	13(37.2%)	5.5455	1	0.3980(medium)	0.0185*
	High	11 (31.6%)	3 (8.6%)				

With respect to the correlation between OVOL1 expression and TNM staging, we observed low reactions among all of the CXPA in stages IVA, most of the stages III and II of the studied CXPA cases. Meanwhile, in stage I, there was an equal distribution between low and high expression. Based on the statistical analysis, there was no statistically significant difference observed between OVOL1 expression among the studied CXPA regarding the clinical stages (χ² = 3.6601, p = 0.30). Despite the lack of statistical significance, the analysis revealed a moderate-to-strong association between the two variables (V = 0.43). This effect size suggests that the nonsignificant p-value is likely attributable to the low statistical power inherent in a small sample size (Table 6).

**Table 6:** Correlation of OVOL1 expression with the TNM staging of carcinoma ex pleomorphic adenoma cases CXPA: carcinoma ex pleomorphic adenoma; OVOL1: ovo-like transcriptional repressor 1; TNM: tumor, lymph node, and metastasis; χ²: test statistic for association; *: significant difference (p < 0.05); N: total number of cases (a) p-value obtained using the Fisher-Freeman-Halton Exact Test due to low expected cell counts (<5) violating Chi-square assumptions. The χ2 statistic and Cramér’s V are reported as the conventional measures of effect size

TNM staging	OVOL1 expression	CXPA (N = 20)	χ²	Effect size (Cramér’s V)	p-value
Stage I	Low	1 (50%)	3.6601	0.4278 (medium-large)	0.30 (a)
	High	1 (50%)			
Stage II	Low	3 (75%)			
	High	1 (25%)			
Stage III	Low	5 (83.3%)			
	High	1 (16.7%)			
Stage IVA	Low	8 (100%)			
	High	0 (0 %)			

## Discussion

The risk of malignant transformation of PA to CXPA increases over time. In the present study, CXPA cases were subclassified according to the WHO Classification of Head and Neck Tumors, 5th Edition, into EMC ex PA (six cases, 30%), MC ex PA (four cases, 20%), SDC ex PA (seven cases, 35%), and Adeno-NOS ex PA (three cases, 15%). These findings were closely aligned with the distributions reported in previous studies by Hu et al. [12] and Tondi-Resta et al. [13]. Although minor variations in the relative frequencies of each subtype were observed, several factors may underlie such discrepancies across studies. In addition to differences in sample size, geographic population, or histopathological assessment criteria, variations in genetic alterations, ethnic background, and even interobserver diagnostic variability have been suggested as possible contributors. Nevertheless, it is worth noting that the current study was based on a relatively small, single-center cohort, which may not fully reflect the broader population-level distributions. Therefore, future multicenter studies with larger, more diverse patient cohorts are needed to confirm and generalize these observations.

As regards OVOL1 expression, several studies have reported its role as a transcription factor that regulates gene expression during various differentiation processes [14-17]. In the present study, all assessed PA and CXPA cases demonstrated immunopositivity for OVOL1, with low and high expression levels. This finding is consistent with previous studies suggesting that OVOL1 may play a dysregulated role in several types of cancer [14,16,18,19].

Interestingly, 12 cases (60%) of PA in our study exhibited high, diffuse cytoplasmic and/or membranous expression for OVOL1, whereas the majority (17 cases; 85%) of CXPA demonstrated low expression. Additionally, there was a statistically significant difference in OVOL1 expression between the studied PA and CXPA (p = 0.0032), with a strong association (effect size = 0.5). These findings indicate that OVOL1 may have potential utility as a diagnostic marker in differentiating PA from CXPA; however, further validation in larger cohorts is warranted.

Our findings are consistent with those of Ito et al., who performed their study on epidermal malignancy of Bowen’s disease. They reported that OVOL1 expression was high in Bowen's disease but markedly reduced in squamous cell carcinoma (SCC). They further concluded that OVOL1 is significantly downregulated in cutaneous SCC [17].

In another study, OVOL1 was identified as a potential diagnostic marker for differentiating normal ocular conjunctiva from SCC. The authors reported that lower OVOL1 scores were associated with disease progression, suggesting its value in distinguishing pre-invasive from invasive SCC [10].

In the current study, low OVOL1 expression was observed in the majority of EMC ex PA, MC ex PA, and SDC ex PA cases, showing a statistically significant difference (p = 0.0354) and a strong association (effect size = 0.63) with the CXPA subtype. In contrast, cases of Adeno-NOS ex PA exhibited high OVOL1 expression in low and intermediate-grade tumors, whereas low OVOL1 reactivity was exclusively observed in high-grade tumors. These results may suggest a contributory role of OVOL1 in modulating tumor aggressiveness and support its possible utility as an adjunctive diagnostic and prognostic marker for distinguishing CXPA subtypes and evaluating tumor behavior.

Similar findings have been reported in breast [14,16] and ovarian carcinomas [11], where OVOL1 downregulation correlated with higher histologic grade and more aggressive phenotypes. This may support the hypothesis that loss of OVOL1 may contribute to dedifferentiation and increased malignant potential.

In the present study, low OVOL1 expression was observed in all CXPA cases with minimal capsular invasion and in six cases (40%) of those with wide capsular invasion. The statistically significant association and strong effect size (p = 0.005, V = 0.5, respectively) observed between OVOL1 expression and capsular invasion patterns suggest a potential link between this marker and specific mechanisms of tumor aggressiveness, rather than a simple binary role.

These findings warrant further consideration, as the distribution reveals a complex pattern, with the complete absence of low OVOL1 expression in noninvasive tumors may indicate that high OVOL1 activity is maintained during initial tumor development before visible invasion. Conversely, 100% of low OVOL1 expression observed in minimally invasive tumors might be associated with an initial physical breach of the capsule, potentially reflecting a temporary invasive phenotype characterized by OVOL1 downregulation. Furthermore, the prevalence of high OVOL1 expression in widely invasive tumors (60%) could suggest that OVOL1 is either re-expressed or retains its prominence during established, widespread invasion.

This pattern strongly suggests that OVOL1 function is related to the cyclical process of EMT and its reversal, MET. Given that OVOL1 is recognized as an EMT suppressor and a promoter of MET, its observed downregulation in the minimal invasive group might temporarily facilitate the necessary mesenchymal shift for the initial capsular invasion. Conversely, the re-elevation of OVOL1 expression in widely invasive tumors could indicate that it is promoting MET, helping the widely disseminated cells to lose their motile phenotype, stabilize, and successfully colonize the new tissue environment. Therefore, the observed association suggests that OVOL1 may represent a crucial variable worth tracking in tumor invasiveness, with its differential expression hinting at a nuanced regulatory role in the dynamics of capsular invasion, dissemination, and colonization.

Similar findings have been reported in oral SCC neoplasm [19] as well as breast carcinoma [14,16], ovarian carcinoma [11], and renal cell carcinoma [18], where reduced OVOL1 expression correlates with more aggressive and invasive phenotypes. 

Regarding tumor recurrence, this study evaluated the association between OVOL1 expression and recurrence in PA and CXPA to explore its potential as a predictor of tumor behavior. The analysis did not demonstrate a statistically significant association (p = 0.2). This finding should be interpreted with caution. The small number of total recurrent cases available for this subanalysis (N = 5) significantly reduced the statistical power of the test, making it difficult to detect a true difference, even if one exists. Despite the nonsignificant p-value, the calculated effect size (ϕ = 1.0) indicates an extremely strong practical association in the sample data where high OVOL1 expression was exclusively confined to the PA group (100% of cases) and entirely absent in the CXPA group (0% of cases). This suggests that the lack of statistical significance is likely attributable to sample size limitation, not a lack of biological association.

Conversely, the data for nonrecurrent cases established a statistically significant association between OVOL1 expression and the tumor groups (χ2 = 5.5455; p = 0.0185). This confirms that the expression pattern of OVOL1 is significantly different between the PA and CXPA groups in the absence of recurrence. The effect size of this association (ϕ = 0.3980) indicates a medium-to-strong association. Specifically, OVOL1-high expression was observed more frequently in the PA group (31.6%) compared to the CXPA group (8.6%) among nonrecurrent cases. This suggests that the distinct molecular pathways related to OVOL1 expression differentiate the biological behavior of the two tumor types.

Therefore, OVOL1 expression appears to be a key variable differentiating the PA and CXPA tumor phenotypes, irrespective of recurrence status. The significant findings in the nonrecurrent cohort provide a clear basis for future mechanistic studies targeting OVOL1's role in favorable outcomes. While the association in the recurrent cohort was nonsignificant, the observed strong effect size (ϕ = 1.0) strongly warrants further investigation with larger sample sizes to definitively establish OVOL1's prognostic value during relapse.

These results are consistent with findings in other epithelial malignancies. As in breast carcinoma, higher OVOL1 expression has been correlated with prolonged relapse-free survival [16]. Similarly, in oral SCC, OVOL1 overexpression suppressed EMT-related invasion and metastasis [19]. These findings suggest the potential role of OVOL1 as both a diagnostic and prognostic biomarker, with lower expression marking a shift toward more aggressive disease phenotypes.

In the present study, OVOL1 expression showed a decreasing expression with advancing clinical stage in CXPA, with all stage IVA cases and most stage III and II cases exhibiting low expression levels, while stage I cases demonstrated an equal distribution between low and high expression. The statistical finding of no significant association between OVOL1 expression and TNM staging (p = 0.300) should be interpreted cautiously, particularly given the small total sample size (N = 20) and the moderate-to-strong effect size (V = 0.4278) observed. The lack of statistical significance likely reflects the low statistical power of the test rather than a true absence of biological association.

A pronounced progressive downregulation trend was evident across advancing TNM stages. Specifically, the proportion of tumors exhibiting high OVOL1 expression decreased sharply from 50% in stage I tumors to 0% in stage IVA tumors. This pattern indicates a potential shift toward the downregulation of OVOL1 as the disease progresses to the most locally advanced stage. OVOL1 is known as a suppressor of the EMT. Its observed downregulation in stage IVA tumors aligns with the expected aggressive phenotype necessary for advanced tumor progression and potential metastasis. This downregulation may facilitate the cancer cells' acquisition of the mesenchymal traits required for wide local dissemination, which characterizes stage IVA.

This is consistent with previous reports in other malignancies linking reduced OVOL1 expression to higher clinical stages and more aggressive disease behavior. In renal cell carcinoma, low OVOL1 expression was significantly correlated with advanced TNM stage, poorer prognosis, and served as an independent prognostic factor [18]. While in oral SCC [19] and ovarian carcinoma [11], downregulation of OVOL1 paralleled disease progression and metastatic potential. These findings collectively support the hypothesis that OVOL1 may play a suppressive role in tumor progression, potentially through inhibition of EMT, and that its reduction might be a marker of advanced disease stage. In the current study, although the exact p-value failed to cross the threshold of significance, the strong effect size and the biological gradient observed suggest that OVOL1 downregulation is a clinically relevant event in the progression to advanced disease stages. Future studies utilizing larger patient cohorts are warranted to confirm this hypothesis and investigate OVOL1's potential as a prognostic marker for late-stage tumors.

Nevertheless, this study has certain limitations that should be acknowledged. The relatively small sample size and single-center design may restrict the generalizability of the findings. Given the rarity of CXPA, the number of available cases was inherently limited, which warrants caution in extrapolating the results. Moreover, the absence of a universally standardized and validated immunohistochemical protocol for OVOL1, as it remains a relatively novel marker, may affect comparability across different studies. Furthermore, the lack of more comprehensive outcome analyses, such as survival or prognostic modeling, restricts the depth of prognostic insights. Future large-scale, multicenter investigations with standardized methodologies are warranted to validate and further expand upon these preliminary observations.

Despite these limitations, to our knowledge, this study represents one of the first to comprehensively assess OVOL1 expression in both PA and CXPA, incorporating histopathological subtypes, tumor grade, stage, capsular invasion, and recurrence. The use of a consistent semiquantitative scoring system, supported by digital image analysis, enhanced reproducibility and comparability of results. In addition, integrating clinicopathological correlations provided valuable insights into the potential diagnostic and prognostic relevance of OVOL1 in salivary gland tumors. While these findings should be interpreted with caution due to the modest cohort size, single-center design, and the lack of standardized protocols and survival analyses, they nonetheless highlight the promise of OVOL1 as a supportive marker for distinguishing PA from its malignant counterpart, CXPA.

## Conclusions

In conclusion, our findings indicate that OVOL1 expression is more consistently retained in benign PA compared to malignant CXPA, with a gradual reduction observed in higher histological grades and more advanced stages. This expression pattern suggests a potential diagnostic utility for OVOL1 in the distinction between PA and CXPA, particularly in morphologically challenging cases, and a possible prognostic relevance in relation to tumor aggressiveness and malignant transformation.

Although these observations are preliminary and constrained by the modest cohort size, they may offer an initial framework for exploring OVOL1 as a potential supportive diagnostic and prognostic biomarker. Further studies, including larger multi-institutional cohorts and mechanistic investigations, are needed to validate these preliminary results and to better establish the potential clinical applicability of OVOL1 in salivary gland tumors.
